# If you build it, they will come…or not. Considerations for women's health in the post-pandemic era of digital innovation

**DOI:** 10.3389/fpubh.2023.1228212

**Published:** 2023-10-13

**Authors:** Martina Anto-Ocrah, Mercy Asiedu, Simrun Rao, Lindsey DeSplinter, Stefanie Hollenbach

**Affiliations:** ^1^Department of Medicine, Division of General Internal Medicine, University of Pittsburgh School of Medicine, Pittsburgh, PA, United States; ^2^Google, Mountain View, CA, United States; ^3^Massachusetts Institute of Technology, Cambridge, MA, United States; ^4^University of Rochester, Rochester, NY, United States; ^5^Department of ObGyn, School of Medicine and Dentistry, University of Rochester, Rochester, NY, United States

**Keywords:** digital health, women's health, FemTech, Africa, culture and information technology (IT)

## Introduction

Culture has been defined as “an internalized and shared framework … through which both the individual and the collective experience the world” (1). Cultural processes shape social institutions, and mold—while in turn being molded by—members of a given cultural or subcultural group (1). The norms that are created by culture can have important implications for health outcomes. Take for example, the case of female genital mutilation, the “cultural” practice of partially or totally removing external female genitalia for non-medical reasons (2). This cultural practice, recognized by some as normal (3), has been associated with several obstetric and gynecological pathologies, and now recognized by the World Health Organization as a violation of human rights (2). This practice, deemed “normal” in one realm of society, is utterly unacceptable in another, and has sparked controversial clashes of belief systems and medical dilemmas which have been widely documented (4–6). At the root of these controversies however, is the fundamental question of “what does pathology mean to a group of people?” At what point does a biochemical change that progresses to pathophysiological change, translate to care-seeking? What forms of care-seeking do people engage in, and what are their reasons for choosing one care-seeking model over another? Are they financial? physical/geographic/infrastructural? (mis)trust? familiarity? racial/gender/cultural discordance? (7).

“Health” is defined by the WHO as “a state of complete physical, mental, and social wellbeing, and not merely the absence of disease or infirmity” (8). While health is sometimes interchanged with wellness, “wellness” is distinctly defined as pro-activity toward good health, and is “an active pursuit of activities, choices and lifestyle that lead to a state of holistic health” (9). Wellness, even more so than health, is highly subjective; and contextualized understandings of relevant wellness metrics and outcomes are important to understand. Does “wellness” mean the same to everyone, and if not, how does the notion of “wellness” differ by various demographics such as age, gender, race/ethnicity/cultural background, socio-economic status, and their intersectionalities? Digital health applications (apps) may cut across components of both health and wellness (10). These include multiplatform (web-based, native computer and smartphone-based, and basic mobile phones) components in health Information and Communications Technology (ICT), quantified self-care and wellness apps, gamification, metadata, sensors and wearable healthcare, electronic health records and medical imaging, telemedicine and personal genomics (10). These apps, when used as interventions, have been successful in high-income countries. However, they have had limited success in low-and middle-income countries (LMICs), and among marginalized populations in high-income countries, even when they are provided at little to no cost (11–14).

**Figure 1:**
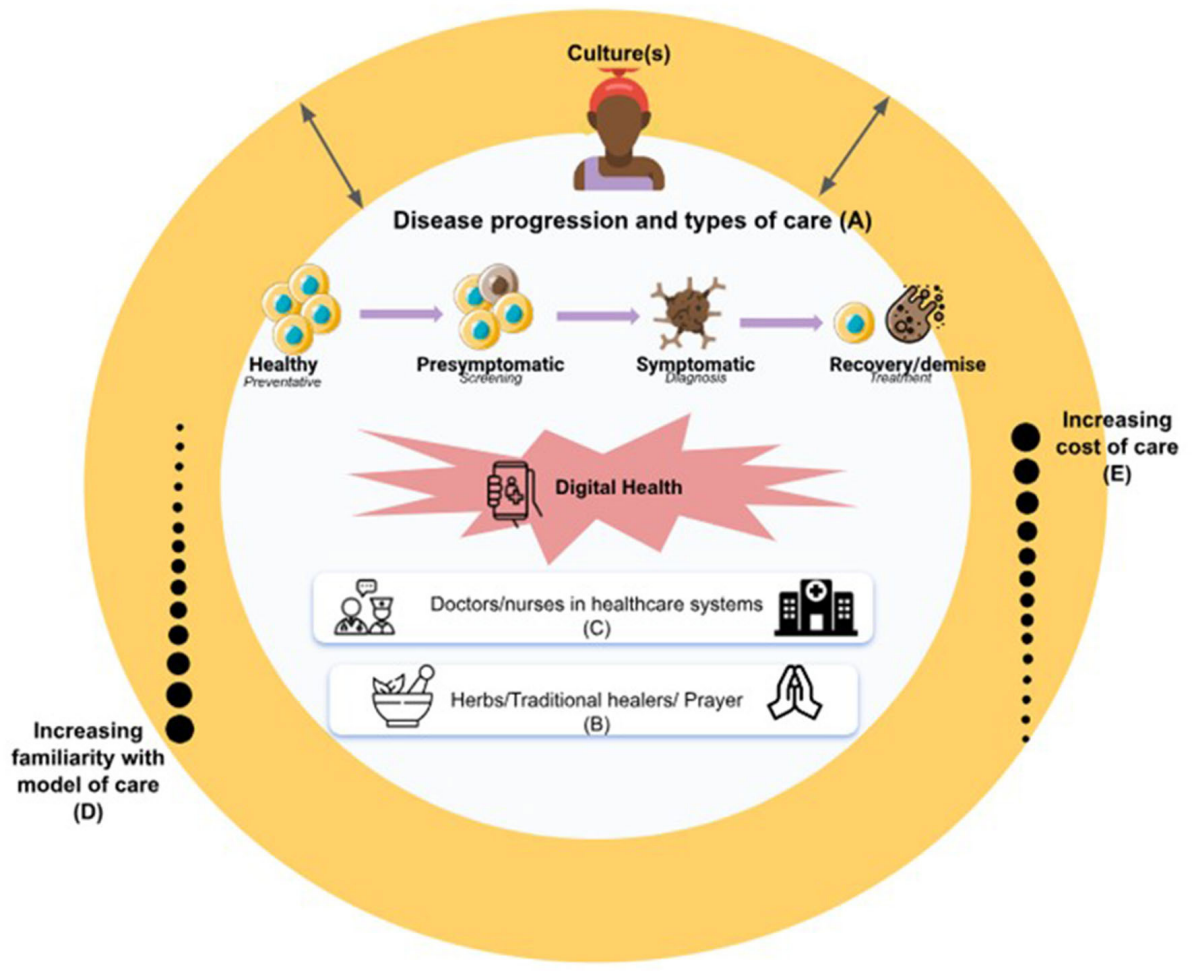
Considerations for digital innovators in achieving the goal of *AI for social good* (15). The availability of digital health tools gives individuals the opportunity to interact with care providers at any point during their pathology/disease progression- from the onset of laboratory diagnosis, to the acute and chronic phases of the disease (A); with the ultimate goal being to prolong optimal health and wellness as much as possible. Cultural normalization may impede a patient's acknowledgment of disease/pathology or “unwellness”. Once acknowledged, treatment and care-seeking plans may involve “culturally acceptable” and familiar norms of care seeking that compete with digital solutions and include prayer (free), herbal remedies and traditional healers (affordable and familiar), and in-person care at medical facilities (less affordable but familiar to the population despite the transportation and geographic barriers) (B–E). Digital adoption requires acculturating people to digital health as another care-seeking option so it becomes familiar to the individual and their community (D).

**In this era of democratization, without considering and understanding what the notion of health- as it relates to “disease” pathology- or “wellness” means to a group of people, digital health and wellness platforms risk falling short of their potential**. Using Figure 1 as a guide, this article outlines considerations that should be taken into account as design anthropologists and developers take on the “social good” agenda of increasing digital access to a critical mass of people globally (15). We discuss notions of disease, wellness, care seeking decisions, competitors and acculturation across different cultures, offering digital health scientists some for food for thought in this post-pandemic era of digital innovation; particularly in women's health. We summarize the key points in Table 1, and conclude the commentary with recommendations for digital entrepreneurs to consider, on their paths to innovation.

**Table 1:** Similarities and differences in key factors affecting digital health innovations in Western and non-Western contexts.

	**Similarities**	**Differences**	**The challenge for digital health innovation**
Disease	“Disease” is generally understood as a pathological process that begins subclinically and if undetected/untreated could result in mortality.	In most resource-rich settings, greater access to preventative care services may “normalize” early disease detection whereas in cost prohibitive settings, “disease” may be recognized only when associated with debilitating symptomatology. Thus “normalization” of disease may be mediated by out-of-pocket expenses/extent of insurance coverage	Understanding that a one-size-fit-all approach may not work, and tailoring digital interventions to fit different notions of “disease” across cultures. E.g., Women in non-Western cultures may be less likely to own their own smartphone, purchase data, or access healthcare without the consent of their partners or in-laws (as dictated by cultural norms) compared to those in Western/resource-rich settings (16)
Wellness	Wellness is a subjective construct of taking care of one's self beyond a “diseased” state, and striving to live a fulfilled life.	“Wellness” is widely accepted in Western contexts as encompassing more than physical health and extending to mental, sexual, reproductive wellness, etc. In non-Western contexts, wellness is often limited to physical activity; with other aspects lagging behind.	Getting buy-in into the wellness revolution; given its subjectivity.
Care seeking behavior	Both contexts have health systems through which patients can seek care, and patients in both contexts do seek care, albeit at different time points. Care-seeking behaviors in both contexts, are driven by cost/insurance coverage/out-of-pocket expenses	In Western contexts, care-seeking is often an individual decision whereas in many non-Western contexts, the decision to seek care is made not by the diseased person solely, but in conjunction with their social institutions and community.	Integrating a “community” approach to the digital health care-seeking decision tree
Competitors, acculturation, and sustainability	Both contexts face issues with sustainability particularly pertaining to cost.	In non-western contexts, traditional, herbal and spiritual medicine, in addition to community-centered care provide sought-after alternatives to clinics and hospitals. In Western contexts clinics and hospitals offer primary sources of care. Pertaining to cost, in LMICs in non-Western contexts, financial burdens exist that impact investments in health and sustainability of health solutions. This is less so in Western contexts	Understanding that herbs and traditional forms of treatment, mixed with spiritual beliefs are at little or no cost to the non-Western populace, and proving that any digital innovation is superior to these.

## Disease

The conceptualization of disease begins with an understanding of an individual or a society's interpretation of **what constitutes a diseased or pathological state that warrants care-seeking**; not merely the diagnosis of “disease” alone. As George Engel (1, 17), author of the biopsychosocial model of care proffers, “it is not necessarily because an individual has been diagnosed with a disease by a physician [or a laboratory examination] that that person [acknowledges that they are indeed sick], feels sick or is considered sick by their environment” (1, 18). In most resource-rich settings, insurance coverage and easier access to preventative care services (beginning in pediatrics), has “normalized” annual physical examinations, mammograms and other preventative services, laboratory services, and frequent patient/provider interactions (7). However, in settings where cost is prohibitive, a biochemical change (indicated by a laboratory exam) must be associated with debilitating symptomatology and/or a sense of urgency before a person may be prompted to seek care (7). This could be further exacerbated by other barriers to care, such as travel time, transportation costs, and long wait times at hospitals (7). Patient/provider interactions may be infrequent, only utilized in emergency situations. While the advent of digital health in resource-limited settings may represent *a new healthcare ecosystem that is unfamiliar to the populace*, the availability of such digital health tools, give individuals the opportunity to interact with care providers at any point during their pathology/disease progression (depicted as “A” in Figure 1); from the onset of laboratory diagnosis, to the acute and chronic phases. The ultimate goal being to prolong “optimal health” as much as possible.

## Wellness

First there is a need to differentiate disease prevention from wellness and wellness related activities. While disease prevention refers to efforts to stem occurrence and severity of a disease, wellness refers to *active efforts on the part of the individual to make choices for a healthy and fulfilling life* (9, 19). Wellness is meant to be holistic, involving physical fitness, nutrition, stress management, and environmental sensitivity (19). Given that wellness is a process toward all-encompassing health, it is even more subjective, and culturally specific than disease prevention. Several digital health tools like wearable fitness trackers, nutrition and dietary managements, stress management apps, and reproductive wellness apps have been—while potentially more accessible than traditional disease prevention tools—developed with the western context of wellness in mind, without much regard to how wellness is perceived in non-western cultures. For instance, what does “mental health wellness” mean in a culture where mental health is dismissed as a curse, or what does “reproductive wellness” mean in a culture were topics around sexuality are taboo? While digital health tools for physical activity are increasingly gaining popularity in non-Western settings, they are yet to extend beyond a small subset of the population and beyond exercise to other wellness areas (20).

### The wellness journey

In Western contexts, wellness journeys typically work in the following stages (9, 19): (1) an acknowledgment that one is unhappy with their current state of wellbeing, mental, physically, emotionally or spiritually. (2) Focus and plans are developed for short or long term mitigation, via hiring an expert and/or utilizing a digital health solution. (3) Tracking of some outcome metric over time through surveys, weight checks or changes in resting heart rate, and long term outcomes such as lower occurrences of disease and a holistic improvement in one's state is noted. The Ayurvedan wellness revolution taking place in the West serves as a great example (21, 22). Ayurveda, an ancient holistic health system originating from India, emphasizes the balance of mind, body, and spirit through personalized lifestyle practices, including dietary choices, herbal remedies, and mindful activities. This wellness approach aligns with the Ayurvedic principle of individual constitution or “dosha”, which categorizes people into different mind-body types, guiding their wellness routines accordingly (22). As Western societies increasingly embrace holistic approaches to health, Ayurveda has gained traction as an alternative way to achieve overall wellbeing and prevent disease by fostering harmony within the individual (21).

In several cultures in Africa however, normalization of “unwellness” or a different perception of what wellness is (compared to the West) may impede a patient's acknowledgment of disease/pathology (7, 9, 19, 20). In cases where these are acknowledged, treatment options may involve religious or traditional solutions (e.g., herbs) that individuals are more familiar with, and/or are less costly than digital solutions (B–E in Figure 1). If a pathology is found in the process, the complex avenue of care seeking decisions, further detailed below, may come to play.

## Care seeking decisions

The idea of digital health is modeled after the Western, individualistic model of care (further enforced by HIPAA laws), which centers disease as an individual experience. In many parts of the world however, the decision to seek care is one that is made not by the diseased person solely, but by their social institutions [partners/spouses, extended family (e.g., in-laws), or community] (7). It is important to note that a community is not a person and a person is not a community. Developing innovations for pregnant American women for example, should consider use cases by racial/ethnic background, as maternal mortality risks for Black women in the United States (US) is much higher than it is for their Caucasian peers, and worsens with acculturation due to social adversity (13). It is imperative to consider more nuanced backgrounds and target populations and incorporate community voices from those targets into any digital health innovation.

Any social good algorithm (15) must be inclusive, sensitive to, and respectful of all parties involved in the care-seeking decision tree of the digital tool. This is a critical step in the digital adoption process. If a patient chooses to seek “modern” forms of care in the healthcare setting (C in Figure 1), it is not only their personal experience that will shape future utilization, but the interactions that their partner, extended family, and community have with the physicians, nurses, and other care providers will also determine future utilization. The patient, and all parties involved in their care, must trust this “new” digitally-based model of care enough to deem it worthy of adoption. This trust-gaining experience is crucial for the (economic) sustainability of several digital interventions, and is the first step in the adoption and acculturation of the digital intervention for the individual's needs.

## Competitors, acculturation, and sustainability

For a person to begin using healthcare digital technology the way it is intended for health (e.g., telehealth intended to bridge patient/provider gap by reducing time, transportation and geographic barriers to care) and wellness (e.g., using wearables to suggest physical activity or stress control), developers must (1) as discussed, first understand how people define and conceptualize disease and wellness (2) gain the user's initial trust then (3) acculturate the people to digital health as another care-seeking option (D in Figure 1). Developers must recognize that prior to this acculturation, they may be competing with other “culturally acceptable” and familiar norms of care seeking, that may range from prayer (free), herbal remedies and traditional healers (affordable and familiar), to in-person care at medical facilities (less affordable but familiar to the population despite the transportation and geographic barriers) (23, 24). For example, in a cross-cultural exploration of COVID-19's impact on antenatal healthcare-seeking behaviors in Ghana and the United States (US) (25), we asked a group of pregnant Ghanaian women if they would accept a telehealth appointment (over in-person) if offered. They all stated that despite the excessive measures they had taken to reduce their COVID exposure, they would forgo the telehealth option (25). As stated by a participant:

“*If they are going to check on me via the telephone, how will they assess me? There are times I feel pain, abdominal pain, side pain, you go and complain and they take a look at it…Sometimes a scan is performed...If you stay home [and opt for telehealth], you wouldn't have access to the scan and all that stuff. I think I prefer going in [person]”* (25)

Their US counterparts were no different. Most of the pregnant women we interviewed expressed skepticism about telehealth: *it “just doesn't feel the same”* (25) stated a participant who voiced concern about the quality of care they would receive via telehealth, citing her lack of familiarity with the shorter, less-structured, and less intimate virtual visits (25).

**In this era of rapid growth, digital health must convince the “naive user” of its utility, capabilities, cultural appropriateness, affordability, and overall fit in their own, personalized conceptualization of disease/pathology/wellness**, and must aim to understand barriers to the form, function and deployment methods of digital health tools, in order to develop culturally specific solutions (26–28).

## Culture and digital health, real world examples

Concrete evidence of the impact of culture on digital health platforms in women's health can be drawn from countries such as Bangladesh and India, where, in an effort to improve maternal health and wellness, Grameen Intel Social Business Limited designed a piece of wearable technology called the COEL bangle (which stands for Carbon Monoxide Exposure Limiter), a smart wristband that is designed to resemble a piece of jewelry commonly worn by women in this region (16, 29, 30). Despite its unassuming exterior, the bracelet features a built-in speaker that educates women about their pregnancy by playing a series of pre-recorded messages in their local languages about diet and nutrition, prenatal environmental hazards in their vicinity, as well as antenatal appointment reminders. Designed with considerations of sociocultural and gender norms in rural Indian communities, the bangle was intended to give women more autonomy as a standalone wearable rather than a mobile app; as norms dictate that women seek permission from male family members or in-laws, in order to have mobile phone access. Thus the COEL bangle does not need to be paired with a smartphone, nor does it need internet connectivity to function. It also has a 10 month battery life (the span of a full-term pregnancy), and can be recharged for use postpartum.

MomConnect (31–33) is another maternal health innovation that was successfully designed and deployed with culture in mind. MomConnect (31) is a cell phone based technology, rolled out nationally by the South African National Department of Health to support maternal health via cell phone messaging. The innovation allows end-users to conveniently receive, access to shareable pregnancy-related educational information. Thus, information is not only accessible to the end-user, but also to whomever the end-user may want to share it with, such as the baby's father, their families, friends, or other mothers. The technology is free of charge to the user, making it more equitable and accessible to individuals of varying socioeconomic statuses across South Africa. MomConnect operates in the 11 official languages of South Africa, and has accumulated almost 2 million registered mothers across the country (31–33). The initiative was developed with key stakeholders and integrated into maternal and child health services already in place on a national scale. It has been lauded as a success story in the tale of digital health, women's health, and cultural adaptations.

Such innovations in women's digital health, designed with a culturally-tailored lens that ranges from the everyday bangle worn by the women, to language adaptations, are necessary to convince the end user of its utility and overall beneficence. The rapid adoption of the COEL bangle and MomConect demonstrate that they were created with not only a user-centered design, but also a culturally-centered framework. Unfortunately, both soon faced threats to their long-term sustainability and scalability due, mostly, to financial barriers. The bangle, for example runs between $12 and 15 US dollars, when the income of the target user is no more than $5 US Dollars a day (16). In the MomConnect model, the cost is absorbed by the service providers rather than the end user, making it free of charge for all end users (and their networks). However, the system requires about $1 million US dollars annually to maintain (33); an expense that is currently funded through public-private partnerships between South Africa's National Department of Health and private companies, including philanthropic donors, which are not guaranteed long-term (33). Thus both the COEL bangle, and MomConnect, lauded for their innovative, user-centered and cultural approaches to women's digital health, are challenged not by user adoption or engagement, but by financial barriers.

**Figure 2:**
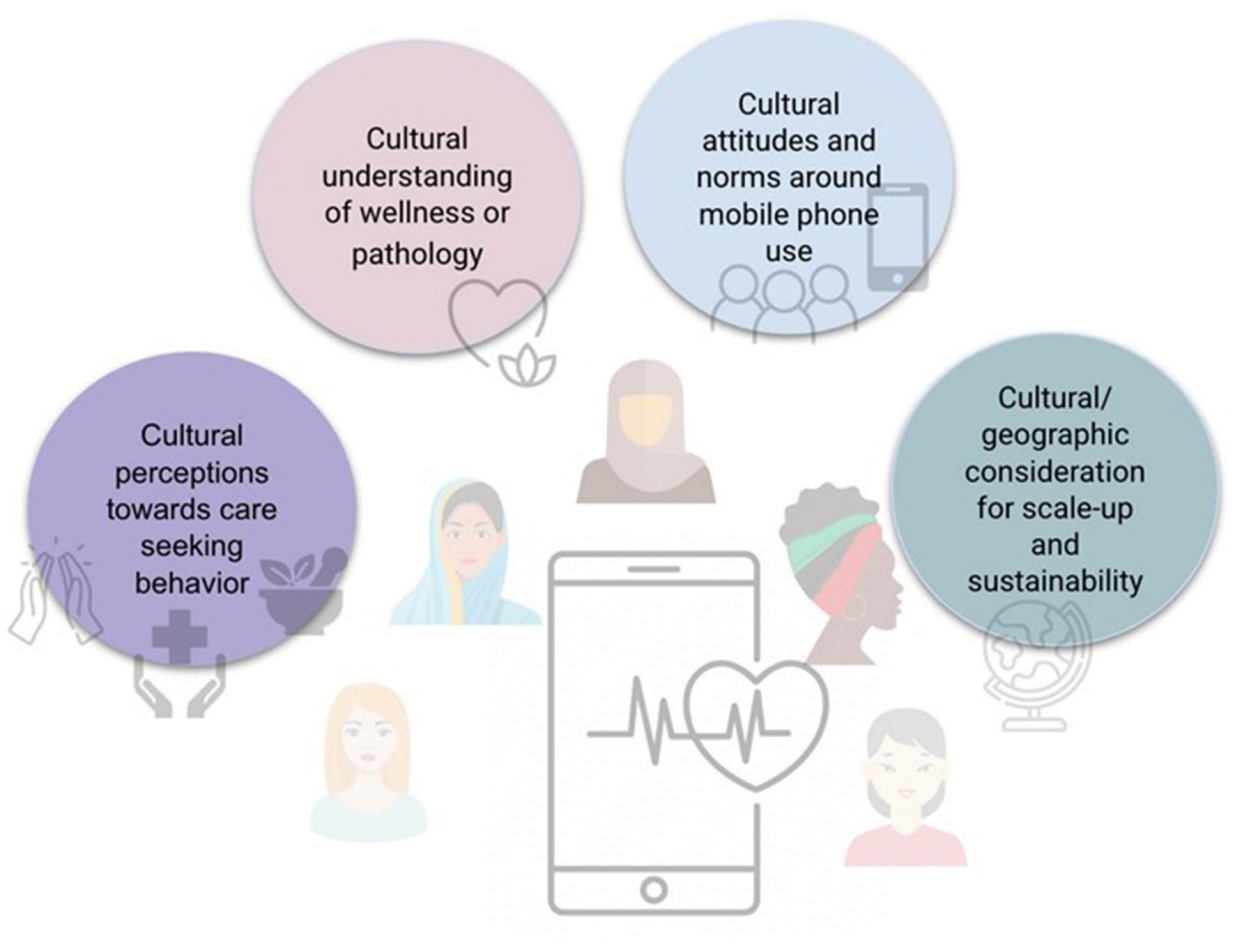
Recommendations and factors to consider for culturally-specific digital health interventions.

If governments are invested in the public's health, if they are invested in decreasing maternal morbidity and mortality, invested in reaching the Sustainable Development Goals of gender equality (Goal 5), good health and wellbeing (Goal 3), reduced inequalities (Goal 10), and sustainable communities (Goal 11), then, they must invest in women's digital health technology (34, 35). A self-sustaining digital model that considers (Figure 2) (26–28) cultural perceptions toward care seeking, cultural understanding of disease and wellness, cultural attitudes and norms around mobile phone use, and considerations for scale-up and sustainability must be designed from the onset.

## Conclusion

Digital inclusion has been deemed a social determinant of health that, if not addressed, can further deepen health disparities (34). The purpose of the World Health Organization's Global Strategy on Digital Health is to promote healthy lives and wellbeing for everyone, regardless of geographic location (35). However, several digital health tools have been developed with Western conceptualizations of disease and wellness, without much regard of how these “states of being” are perceived in non-Western cultures. Even though innovations in the digital sphere are happening at unprecedented speeds, their adoption tends to be slow, their longevity short-lived, and their overall impact on health systems and people's wellbeing, questionable (36–38). As stated by the WHO, “To improve health and reduce health inequalities, rigorous evaluation of eHealth is necessary to generate evidence and promote the appropriate integration and use of these technologies…to ensure that such investments do not inappropriately divert resources from alternative, non-digital approaches” (36). Before we address issues of “digital inclusion,” “digital literacy,” and “digital access” (34), we must first understand what disease and wellness mean to the end user. For if, despite a pathology report, the end user does not deem themselves “diseased” or “unwell”, a readily available laboratory portal, or a same-day delivery pharmacy prescription interface will be deemed useless. Digital innovations for women's health must also consider the socio-cultural norms imposed on women in their respective designs. Can a woman own a mobile phone? If yes, can she buy her own internet data for connectivity? If yes, can she afford the costs over the needs of her family? In many parts of the world, a woman's autonomy lies in the hands of her male partners and/or in-laws, not in her own. Lastly, a self-sustaining digital model-with government and financial stewardship and investment- must be designed from the onset. Plans for scale up and long-term sustainability must involve government buy-in and financial stewardship, lest these innovations, no matter how culturally appropriate they are, will die in their infancy.

## Future work

In this commentary, we identify gaps in the cultural adaptation of digital health tools (Figure 1), and recommend a framework for digital health developers to consider for the development of culturally-specific digital health solutions (Figure 2). This commentary is presented primarily from the perspectives of Ghanaian, Indian, and North American (USA) female clinical, public health, and digital health researchers, whose points of view reflect their own lived and research experiences. Future work would benefit from wider-spread examinations of interviews or focus groups on the adoption of digital health innovations in different cultural contexts.

## Author contributions

MA-O: conceptualization and writing—original draft. MA-O, MA, SR, LD, and SH: writing—review and editing. All authors contributed to the article and approved the submitted version.
